# *DUSP5* and *PHLDA1* mutations in mature cystic teratomas of the ovary identified on whole-exome sequencing may explain teratoma characteristics

**DOI:** 10.1186/s40246-022-00424-w

**Published:** 2022-10-26

**Authors:** Wen-Chung Wang, Yen-Chein Lai

**Affiliations:** 1 Department of Obstetrics and Gynecology, Jen-Ai Hospital, Taichung, 412 Taiwan; 2 Department of Medical Laboratory and Biotechnology, Chung Shan Medical University, No. 110, Sec. 1, Chien Kuo N. Road, Taichung, 402 Taiwan; 3 Clinical Laboratory, Chung Shan Medical University Hospital, Taichung, Taiwan

**Keywords:** Mature cystic teratomas, Dermoid cysts, Whole-exome sequencing

## Abstract

**Background:**

Mature cystic teratomas of the ovary are the most common type of germ cell tumor, comprising 33% of ovarian tumors. Studying these tumors may result in a better understanding of their stepwise developmental processes and molecular bases and provide useful information for the development of tissue-engineering technologies.

**Methods:**

In the present study, 9 mature cystic teratomas of the ovary were analyzed by whole-exome sequencing and the results were compared with the Catalogue of Somatic Mutations in Cancer and dbSNP databases.

**Results:**

Mutations were validated in 15 genes with alterations in all 9 (100%) samples and changes in protein coding. The top 10 mutated genes were *FLG*, *MUC17*, *MUC5B*, *RP1L1*, *NBPF1*, *GOLGA6L2*, *SLC29A3*, *SGK223*, *PTGFRN*, and *FAM186A*. Moreover, 7 variants in exons with changes in protein coding are likely of importance in the development of mature cystic teratomas of the ovary, namely *PTGFRN*, *DUSP5*, *MPP2, PHLDA1*, *PRR21*, *GOLGA6L2,* and *KRTAP4-2*.

**Conclusions:**

These genetic alterations may play an important etiological role in teratoma formation. Moreover, novel mutations in *DUSP5* and *PHLDA1* genes found on whole-exome sequencing may help to explain the characteristics of teratomas.

**Supplementary Information:**

The online version contains supplementary material available at 10.1186/s40246-022-00424-w.

## Background

The term teratoma is derived from the Greek word *teraton,* meaning monster, and was first used by Rudolf Virchow in 1863 [1]. Teratomas are true neoplasms arising from totipotential germ cells [2] and classified as mature or immature, depending on the degree of differentiation of their components [3]. They are defined histologically as containing tissues derived from all 3 germ layers: ectoderm, mesoderm, and endoderm [3]. Extragonadal germ cell tumors represent only 1–5% of all germ cell tumors and frequently present as sacrococcygeal teratomas [4]. One of the most common locations is the ovary, although they also occur in the testes [5]. Ovarian teratomas include mature cystic teratomas (dermoid cysts), immature teratomas, and monodermal teratomas (e.g., struma ovarii, carcinoid tumors, neural tumors) [3]. In the ovaries, most benign teratomas are cystic and referred to in clinical parlance as dermoid cysts [5]. Mature cystic teratomas of the ovary are the most common germ cell tumor, comprising 33% of ovarian tumors [6].

The presence of three somatic germ layers within teratomas is considered the best indicator of the pluripotency of human embryonic stem (hES) cell lines [7, 8]. Studying teratomas may aid in the development of safer hES cell therapies [9]. As developmental processes cannot be investigated in intact mammalian embryos [10], teratomas represent an alternative development model. The arrangement of different tissue types in teratomas in many ways recapitulates organogenesis within the embryo [11]. It is also important to elucidate the stepwise developmental processes and molecular bases of teratomas, as these may provide useful information for the development of tissue-engineering technologies [12]. The genetic and environmental conditions that confer teratoma susceptibility remain poorly understood [13], although mutations in several genes might underlie increased tumor incidence. Limited studies, most of which have been case reports, have demonstrated mutations in mature cystic teratomas [14, 15]. In a recent study, an attempt was made to identify the genomic abnormalities in squamous cell carcinomas (SCCs) arising from ovarian mature cystic teratomas using next-generation sequencing [16]. The most frequently altered genes in SCC are *TP53* (20/25 cases, 80%), *PIK3CA* (13/25 cases, 52%), and *CDKN2A* (11/25 cases, 44%) [16]. The aim of this study was to elucidate the possible etiological roles of genetic alterations identified on whole-exome sequencing (WES) in teratoma formation.

## Materials and methods

### Clinical samples

The Institutional Review Board of Chung Shan Medical University Hospital approved all procedures, and informed consent was obtained from all subjects prior to collecting their genetic material for the study (reference CS19118). Eight 18–46-year-old patients with ovarian teratoma(s) were enrolled, including one woman with bilateral mature cystic teratomas (Tera-10R and Tera-10L) of the ovary [17, 18], totaling 9 samples (Table 1).

**Table 1:** Baseline characteristics of 9 mature cystic teratomas of the ovary

#	Age	Type of operation	Right/left	Size (cm)	Gross content	Microscopic content	Immature element/malignancy
1	32	Oophorocystectomy	Right	5 × 3.5 × 3	Wax-like material and hairs	Squamous epithelium, skin adnexa, bone and intestinal mucosa	No/No
2	35	Laparotomy	Right	11 × 9 × 8.8	Smooth outer surface with waxy material	Squamous epithelium and thyroid tissue	No/No
5	46	Laparoscopic cystectomy	Right	4.7 × 3.8 × 2.8	Wax-like material	Squamous epithelium, skin adnexa, bone and intestinal mucosa	No/No
6	27	Laparoscopic cystectomy	Right	8.2 × 3 × 1.3	Wax-like material	Squamous epithelium, skin adnexa, bone and intestinal mucosa	No/No
7	18	Laparoscopic cystectomy	Right	6.1 × 3.1 × 1.6	Wax-like material	Squamous epithelium, skin adnexa, bone and intestinal mucosa	No/No
9	35	Cystectomy	Right	7.5 × 5.5 × 3	Wax-like material and hair shaft	Multidermal component	No/No
10R	34	Laparoscopic cystectomy	Right	4 × 2.1 × 1	Wax-like material	Squamous epithelium, skin adnexa, bone and intestinal mucosa	No/No
10L	34	Laparoscopic cystectomy	Left	3.5 × 1.5 × 1	Wax-like material	Squamous epithelium, skin adnexa, bone and intestinal mucosa	No/No
11	37	Partial cystectomy and enucleation	Left	6 × 4 × 2.8	Wax-like material and hair shaft	Squamous epithelium, skin adnexa, fat, nerves, mature cartilage and lamellar bone	No/No

### Histological examination

Upon cutting, cystic masses were found to contain fat, hair, and bony tissue. Histological sections were prepared from formalin-fixed paraffin-embedded (FFPE) blocks and stained with hematoxylin and eosin for histopathological review. Microscopically, sebaceous gland, skin appendages, and thyroid follicles were also evident (Table 1). These tumors were considered to be mature without immature components after examination of multiple sections (Table 1). There was no evidence of malignancy (Table 1).

### Isolation of DNA from blood

Genomic DNA was extracted from paraffin-embedded sections of the teratomas with the DNA FFPE Tissue Kit (Qiagen, Hilden, Germany) according to the manufacturer’s instructions. DNA from the teratomas was obtained from solid nodule within the inner site and finally dissolved in 100 μl of TE buffer (10 mM Tris–HCl, pH 8.0, and 1 mM EDTA). DNA concentration of each sample was measured using NanoDrop UV–VIS Spectrophotometer.

### Library preparation and whole-exome sequencing (WES)

WES was carried out at a biotechnology company (Genomics BioSci & Tech, Taipei, Taiwan). A total of 200 ng DNA per sample served as the input material. Sequencing libraries were generated using Agilent SureSelect Human All Exon V6 kit (Agilent Technologies, California, USA) following the manufacturer’s recommendations with index codes added to each sample.

Briefly, fragmentation was carried out by hydrodynamic shearing system (Covaris, Massachusetts, USA) to generate 180–280 bp fragments. Remaining overhangs were converted into blunt ends via exonuclease/polymerase activities. After adenylation of 3’ ends of DNA fragments, adapter oligonucleotides were ligated. DNA fragments with ligated adapter molecules on both ends were selectively enriched via PCR reaction. After PCR reaction, libraries were hybridized with liquid phase using biotin labeled probe. Then, magnetic beads with streptavidin were used to capture the exons of genes. Captured libraries were enriched in a PCR reaction to add index tags in preparation for sequencing. Products were purified using AMPure XP system (Beckman Coulter Inc, California, USA) and quantified via Agilent high sensitivity DNA assay conducted on Agilent Bioanalyzer 2100 system. Libraries were sequenced on Illumina NovaSeq 6000 platform and 150 bp paired-end reads were generated by Genomics BioSci & Tech Co.

### Bioinformatics analysis

Bioinformatics analysis pipeline followed from the sequencing step. Low-quality bases and sequencing adapters in raw data generated from Illumina sequencer were removed using the program Trimmomatic. Subsequently, the reads were aligned to reference genome using Burrows-Wheeler Aligner (BWA) [19]. The results of alignment step were recorded in.bam format. Then, .bam file was processed using Picard-tools with sorting and duplicate marking. After that, variant calling was performed with Genome Analysis Toolkit (GATK) and HaplotypeCaller task and variants were annotated by VEP [20]. Gene sequences were aligned to reference sequences based on human genome build GRCh37/UCSC hg19.

## Results

### Tumor-only whole-exome sequencing

Next-generation WES was performed on 9 teratoma-derived FFPE specimens without matched normal controls that were denoted tumor-only. On average, 6.97 Gb of high-quality clean bases were generated per sample and 99.9% of sequence reads were uniquely aligned with the human reference genome. The depth of on-target coverage of each exome ranged from 15 to 132 with an average of 78.91. In this study, we utilized somatic variant calling with MuTect2 from GATK to detect somatic variants, and then we filter them to obtain a more confident set of somatic variant calls with FilterMutectCalls. Variants identified on WES were compared against the Catalogue of Somatic Mutations in Cancer (COSMIC) and dbSNP databases.

### Spectrum of putative somatic mutations

We extracted the variants, which were annotated as PASS in VCF file and found 38,633 putative somatic mutations, including non-synonymous and splicing mutations, according to dbSNP filtering and COSMIC criteria. There were 26,132 single nucleotide variants (SNVs). Of these, 15,099 SNVs were within exons. Moreover, these variants were functionally annotated and their impact was predicted using WEP software [21]. It was found that 159, 5,561, 7,460, and 1,919 were of high, low, moderate, and modifier impacts, respectively.

**Fig. 1:**
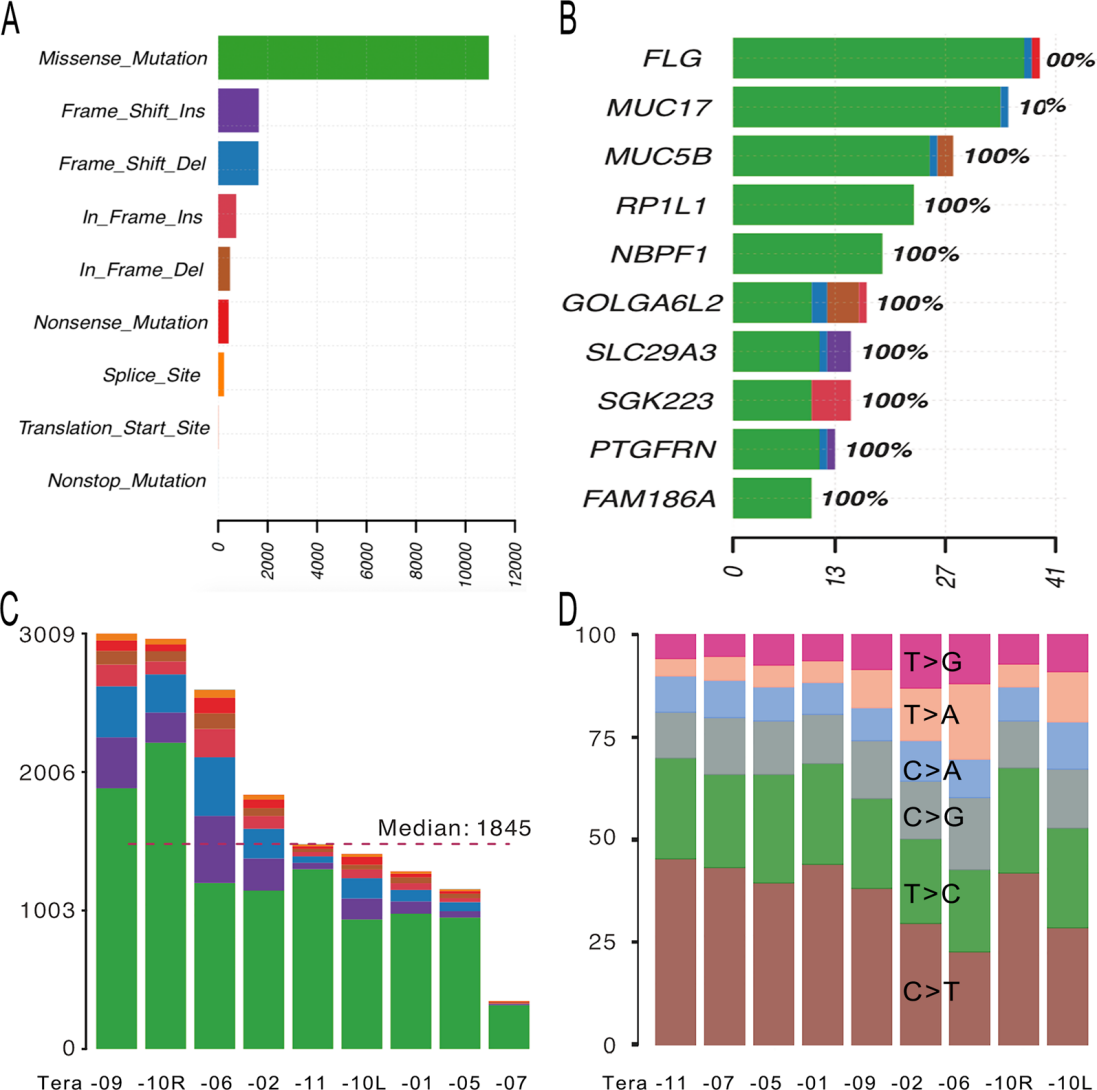
Somatic mutation profiles of mature cystic teratomas of the ovary. **A** Variant classification, **B** top 10 mutated genes, **C** variants per sample, and **D** % of mutation type per sample for 9 mature cystic teratomas of the ovary

**Fig. 2:**
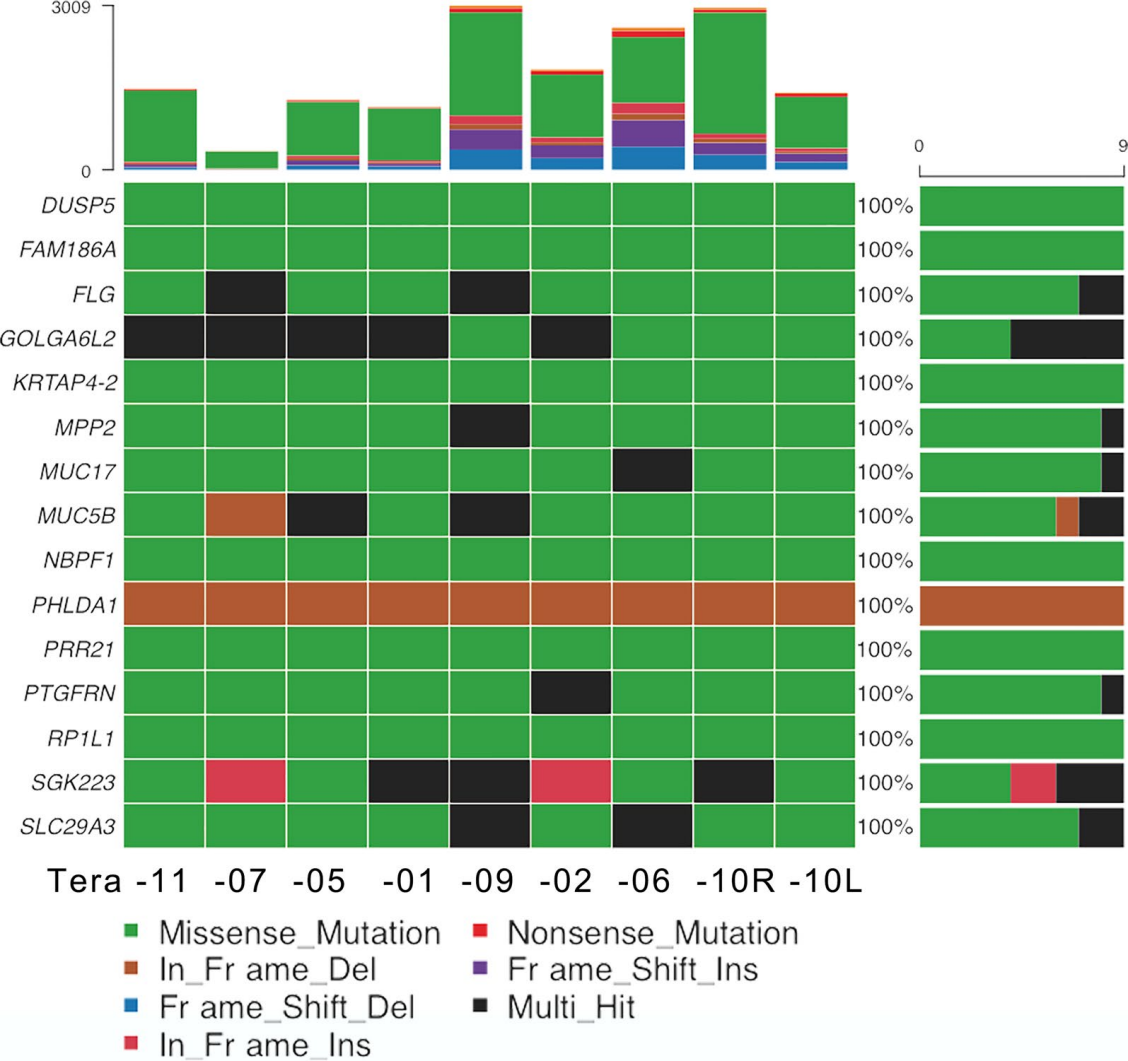
Oncoplot of the 15 most frequently mutated genes with changes in protein coding in teratomas. Each column represents a different sample and each row a different gene. Colored squares represent mutated genes. Mutations are shown according to variant type as indicated in the legend. Genes annotated as “Multi_Hit” have more than one mutation in the same sample. The barplot at the top shows the number of mutated genes for each patient according to mutation type. The barplot on the right presents the numbers of mutated teratomas for each gene according to mutation type

Somatic mutation profiles of the ovarian teratomas are shown in Fig. 1. In terms of variant classification (Fig. 1A), missense mutation was the most common, followed by frame shift insertion, frame shift deletion, in frame insertion, and in frame deletion. Mutations were validated in 15 genes with alterations in 9 (100%) samples and changes in protein coding (Fig. 2). The top 10 mutated genes were *FLG*, *MUC17*, *MUC5B*, *RP1L1*, *NBPF1*, *GOLGA6L2*, *SLC29A3*, *SGK223*, *PTGFRN*, and *FAM186A* (Fig. 1B). Genetic variants detected in exons with a change in protein coding in the top 10 mutated genes are shown in Additional file 1. *DUSP5*, *KRTAP4-2*, *MPP2*, *PHLDA1*, and *PRR21* were added to complete the list of the top 15 most frequently mutated genes. Oncoplot of the 15 most frequently mutated genes with changes in protein coding in 9 ovarian teratomas is shown in Fig. 2.

**Fig. 3:**
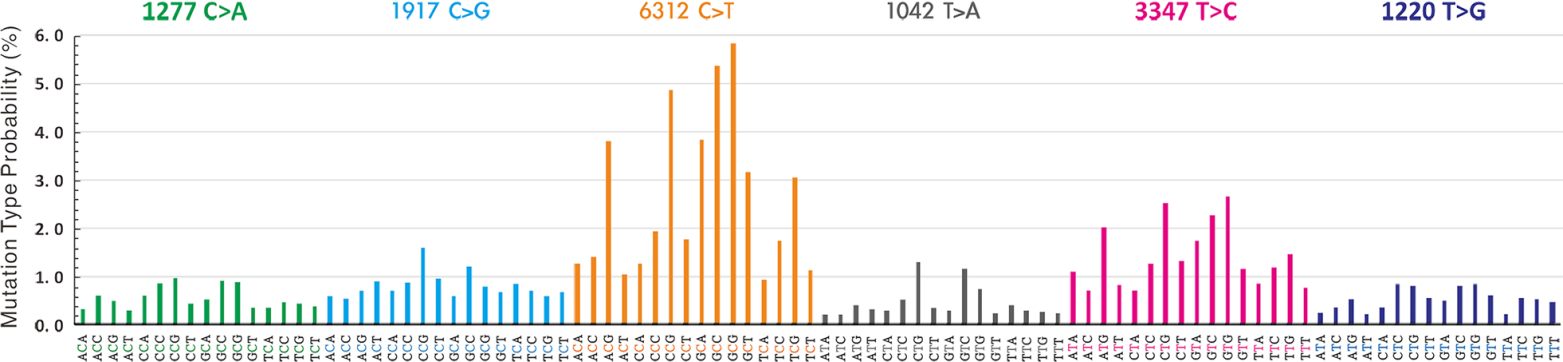
Mutational signature found in mature cystic teratomas of the ovary. Pattern of substitutions for signatures according to the 96 substitutions defined by substitution class and sequence context immediately 3′ and 5′ of the mutated base. Probability bars are shown for the 6 types of substitutions. Mutational signature is based on the trinucleotide frequency of the human genome

The number of variants of each target teratoma of each exome ranged from 347 to 3,009 with an average of 1,845 (Fig. 1C). Among the missense mutations, the most common was C>T, followed by T>C and C>G (Fig. 1D). The patterns of substitutions for each mutational signature are shown in Fig. 3.

### Mutational landscape of teratomas

**Fig. 4:**
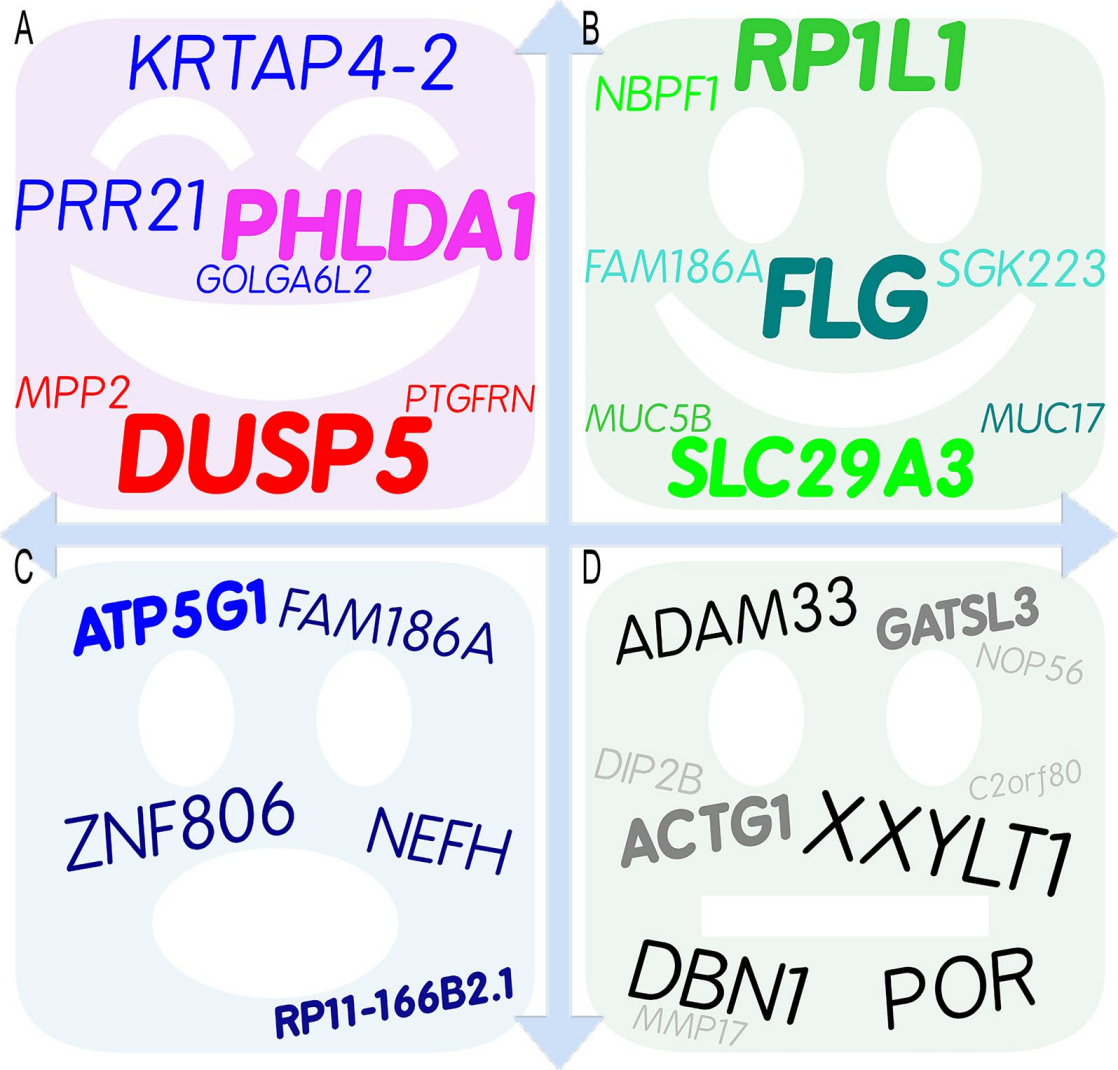
Word cloud artwork illustrates the important genes in ovarian mature cystic teratomas identified on WES. Mutations were validated in 15 genes with alterations in all 9 (100%) samples: 7 variants in exon with changes in protein coding (**A**) and 8 leftovers (**B**). There were 12 variants in exons: 7 variants with changes in protein coding (**A**) and 5 variants without changes in protein coding (**C**). A, 7 variants in exon with changes in protein coding; 3 substitutions are shown in red, 1 deletion is shown in magenta, and 3 SNVs are shown in pink. **C** 5 variants in exon without changes in protein coding; 1 deletion is shown in light blue and 4 SNVs are shown in dark blue. **D** 10 variants in intron without changes in protein coding; 4 substitutions are shown in black, 1 deletion and 1 insertion are shown in dark gray, and 4 SNVs are shown in light gray

Among the prevalent mutated genes, 22 common somatic variants were detected in the 9 paraffin-embedded tumor specimens (Table 2; Fig. 4). Mutations were validated in 15 genes with alterations in all 9 (100%) samples (Fig. 2): 7 genes with the same variant in exon and changes in protein coding (Fig. 4A) and 8 leftover genes with different variants (Fig. 4B). There were 12 variants in exons (Fig. 4A, C) and 10 variants in introns (Fig. 4D). Seven of the 12 variants in exons were associated with changes in protein coding (Fig. 4A): *PTGFRN*, *DUSP5*, *MPP2, PHLDA1*, *PRR21*, *GOLGA6L2*, and *KRTAP4-2*. Three variants were substitutions (shown in red in Fig. 4A) with moderate impact: rs71483896 (c.828_829delinsGA, p.Ser277Thr) in exon 3 of the *PTGFRN* gene on chromosome 1 (missense variant, depth 631, average depth 70.11); rs35834951 (c.658_659delinsAT, p.Ala220Met) in exon 3 of the *DUSP5* gene on chromosome 10 (missense variant, depth 760, average depth 84.44); and rs70964679 (c.240_241delinsGC, p.His80_Val81delinsGlnLeu) in exon 4 of the *MPP2* gene on chromosome 17 (missense variant, depth 727, average depth 80.78). One variant was a three-nucleotide deletion with moderate impact, rs71716769 (c.582_584del, p.Gln204del) in exon 1 of the *PHLDA1* gene on chromosome 12 (inframe deletion, depth 615, average depth 68.33) (shown in magenta in Fig. 4A). There were three SNVs with moderate impact and changes in protein coding (shown in pink in Fig. 4A): rs6732185 (c.1025A>T, p.Lys342Met) in exon 1 of the *PRR21* gene on chromosome 2 (missense variant, depth 1044, average depth 116); rs59122400 (c.949C>T, p.Arg317Trp) in exon 8 of the *GOLGA6L2* gene on chromosome 15 (missense variant, depth 745, average depth 82.77); and rs389784 (c.284A>G, p.Tyr95Cys) in exon 1 of the *KRTAP4-2* gene on chromosome 17 (missense variant, depth 985, average depth 109.44). They were all of moderate impact, such that a non-disruptive variant might change protein effectiveness [21]. Among the 15 prevalent mutated genes, 8 with different variants in exons with changes in protein coding are important for the development of mature cystic teratomas of the ovary: *FLG*, *MUC17*, *MUC5B*, *RP1L1*, *NBPF1*, *SLC29A3*, *SGK223*, and *FAM186A* (Fig. 4B). The variants are shown in Additional file 1. Five of the 12 variants were in exons without changes in protein coding (Table 2; Fig. 3C): a non-coding transcript exon variant of modifier impact in *ZNF806* (depth 224); a variant of modifier impact within the 3′-untranslated region in *ATP5G1* (depth 586); and three synonymous variants with low impact in *RP11-166B2.1* (depth 1122), *CACNA1A* (depth 209), and *NEFH* (depth 1076).

**Table 2:** Common genetic variants detected in all 9 paraffin-embedded tumor specimens on whole-exome sequencing

#chr	Position	Variation	REF	ALT	Depth	SA_AF	Impact	Symbol	E/I	Class	Consequence	HGVS nomenclature	HGVSp nomenclature
*Exon*													
chr1	117487710	rs71483896	AT	GA	631	None	Moderate	*PTGFRN*	3/9	Substitution	Missense variant	c.828_829delinsGA	p.Ser277Thr
chr2	133074851	rs7355688	A	T	224	None	Modifier	*ZNF806*	3/3	SNV	Non-coding transcript exon variant	n.316A>T	
chr2	240981375	rs6732185	T	A	1044	0.08	Moderate	*PRR21*	1/1	SNV	Missense variant	c.1025A>T	p.Lys342Met
chr10	112266822	rs35834951	GC	AT	760	None	Moderate	*DUSP5*	3/4	Substitution	Missense variant	c.658_659delinsAT	p.Ala220Met
chr12	76424937	rs71716769	TTGC	T	615	None	Moderate	*PHLDA1*	1/2	Deletion	Inframe deletion	c.582_584del	p.Gln204del
chr15	23686673	rs59122400	G	A	745	0.67	Moderate	*GOLGA6L2*	8/8	SNV	Missense variant	c.949C>T	p.Arg317Trp
chr16	12021332	rs256390	G	A	1122	0.68	Low	*RP11-166B2.1*	8/8	SNV	Synonymous variant	c.1092C>T	p.Pro364Pro
chr17	39334133	rs389784	T	C	985	None	Moderate	*KRTAP4-2*	1/1	SNV	Missense variant	c.284A>G	p.Tyr95Cys
chr17	41960633	rs70964679	CG	GC	727	None	Moderate	*MPP2*	4/13	Substitution	Missense variant	c.240_241delinsGC	p.His80_Val81delinsGlnLeu
chr17	46973139	rs35074390	TG	T	586	1.0	Modifier	*ATP5G1*	5/5	Deletion	3′ UTR variant	c.*15delG	
chr19	13319693	rs16051	A	G	209	0.21	Low	*CACNA1A*	46/47	SNV	Synonymous variant	c.6657 T>C	p.His2219His
chr22	29885861	rs165923	T	C	1076	0.00	Low	*NEFH*	4/4	SNV	Synonymous variant	c.2232 T>C	p.Ala744Ala
*Intron*													
chr2	209049840	rs10804167	C	T	173	0.68	Modifier	*C2orf80*	2/8	SNV	Intron variant	c.42-84G>A	
chr3	194991220	rs370476236	AA	GC	131	None	Modifier	*XXYLT1*	1/3	Substitution	Intron variant	c.504+63_504+64delinsGC	
chr5	176895817	rs386695380	GC	AA	502	None	Modifier	*DBN1*	3/14	Substitution	Intron variant	c.148+27_148+28delinsTT-	
chr7	75614863	rs71526806	TG	CT	394	None	Modifier	*POR*	12/15	Substitution	Intron variant	c.1399-34_1399-33delinsCT	
chr12	51034721	rs2731436	C	T	186	None	Modifier	*DIP2B*	3/37	SNV	Intron variant	c.301+86C>T	
chr12	132323294	rs4964884	T	C	211	0.80	Low	*MMP17*	3/9	SNV	Splice region variant Intron variant	c.422+8T>C	
chr17	79477898	rs59886367	GC	G	1076	0.03	Modifier	*ACTG1*	4/4	Deletion	Intron variant	c.985-40del	
chr20	2633404	rs6115307	C	G	181	0.00	Modifier	*NOP56*	1/11	SNV	Intron variant	c.4-84C>G	
chr20	3657803	rs71212741	AT	GA	611	None	Modifier	*ADAM33*	2/21	Substitution	Intron variant	c.178-14_178-13delinsTC	
chr22	30681797	rs6147585	T	TCTGCCCCAGCCCTTGGTGCTCCCC	609	None	Modifier	*GATSL3*	8/8	Insertion	Intron variant	c.921+23_921+24insGGGGAGCACCAAGGGCTGGGGCAG	

*chr* chromosome, *REF* reference, *ALT* alteration, *SA_AF* South Asian allele frequency, *E/I* Exon/Intron

Impacts: High, the variant is assumed to have great (disruptive) impact on the protein, possibly causing protein truncation or loss of function or triggering of nonsense mediated decay; moderate, a non-disruptive variant that might change protein effectiveness; modifier, non-coding variant or variant affecting non-coding genes, with predictions difficult or no evidence of impact (https://asia.ensembl.org/Help/Glossary?id=535, accessed September 2022)

Underline: variants in exons with changes in protein coding

Four of the 10 variants in introns were substitutions, resulting in modifier impact without changes in protein coding (Table 2, shown in black in Fig. 4D): rs370476236 (c.504+63_504+64delinsGC) in intron 1 of the *XXYLT1* gene on chromosome 3 (depth 131); rs386695380 (c.148+27_148+28delinsTT) in intron 3 of the *DBN1* gene on chromosome 5 (depth 502); rs71526806 (c.1399-34_1399-33delinsCT) in intron 12 of the *POR* gene on chromosome 7 (depth 394); and rs71212741 (c.178-14_178-13delinsTC) in intron 2 of the *ADAM33* gene on chromosome 20 (depth 611). Four of the 10 variants in introns were SNVs of modifier or low impact: rs10804167 (c.42-84G>A) in intron 2 of the *C2orf80* gene on chromosome 2 (depth 173); rs2731436 (c.301+86C>T) in intron 3 of the *DIP2B* gene on chromosome 12 (depth 186); rs4964884 (c.422+8T>C) in intron 3 of the *MMP17* gene on chromosome 12 (splice region variant, depth 211); and rs6115307 (c.4-84C>G) in intron 1 of the *NOP56* gene on chromosome 20 (depth 181). In addition, rs59886367 (c.985-40del) in intron 4 of the *ACTG1* gene on chromosome 17 (depth 1076) and rs6147585 (c.921+23_921+24insGGGGAGCACCAAGGGCTGGGGCAG) in intron 8 of the *GATSL3* gene on chromosome 22 (depth 609) were deletion and insertion with modifier impact, respectively.

## Discussion

The most common variant was C>T, followed by T>C and C>G (Figs. 1D, 3). Most variants were C>T/G>A, similar to Signature 6 which is characterized predominantly by C>T at NpCpG mutations in the mutational signature analysis by Alexandrov et al*.* [22]. Davies et al*.* reported that *MLH1*-inactivated breast cancers are combinations of predominant mutation types C>T/G>A and T>C/A>G transitions (classified as Signature 6) with overwhelming indel mutagenesis, particularly deletions at polynucleotide repeat tracts [23]. However, only one *MLH1*-inactivated missense variant was observed in Tera-11 in this study: rs63750447 (c.1151T>A, p.Val384Asp) in exon 12.

Except for some case reports, few studies have been published on the mutations in mature cystic teratomas [14, 15]. Point mutations in the *p53* gene and *p16* gene are associated with SCCs that arise in mature cystic teratomas [24]. In a recent study, genomic abnormalities in such SCCs were identified using next-generation sequencing [16]. The most frequently altered genes in SCC are *TP53* (20/25 cases, 80%), *PIK3CA* (13/25 cases, 52%), and *CDKN2A* (11/25 cases, 44%) [16]. However, only one *TP53* missense variant in exon with changes in protein coding was observed in Tera-10R: rs1042522 (c.215C>G, p.Pro72Arg) in exon 4. No *PIK3CA* or *CDKN2A* variants were detected in the 9 paraffin-embedded tumor specimens in this study.

Among the prevalent mutated genes, 7 with the same variant in exons with changes in protein coding are important for the development of mature cystic teratomas of the ovary: *PTGFRN*, *DUSP5*, *MPP2, PHLDA1*, *PRR21*, *GOLGA6L2,* and *KRTAP4-2* (Fig. 4A). *PTGFRN* encodes a 135-kDa protein PTGFRN (Prostaglandin F2 receptor negative regulator) that inhibits binding of PGF2-α to its specific receptor [25]. Also known as CD315, EWI-F, CD9P-1, and SMAP-6, PTGFRN has been shown to interact with CD9 and CD81 [26–28] and is potentially an important regulated protein in the development of the antral follicle. Down-regulation of PTGFRN in GCs may lead to follicular atresia [29]. Differentially expressed genes, especially those in five modules, including *OAS1, IFI27, LPAR1, PTGFR, ITGB4,* and *ITGA6*, might participate in the epithelial-mesenchymal transition process in breast cancer cell line DKTA [30]. *DUSP5* gene encodes dual specificity phosphatases (DUSPs) that inactivate ERK 1/2 through dephosphorylation and inhibit inflammatory gene expression [31]. DUSP5 protein plays an important role in the maintenance of pluripotency in mouse embryonic stem cells and may be required for embryoid body development [32]. DUSP5 promotes osteogenic differentiation through SCP1/2-dependent phosphorylation of SMAD1 [33]. *MPP2* gene encodes palmitoylated membrane protein 2, which is a member of the membrane-associated scaffold protein family known as MAGUKs (membrane-associated guanylate kinase homologs) [34]. *MPP2* is expressed in multiple cell types and plays important roles in cellular proliferation, differentiation and tumorigenesis [35]. MPP2 protein interacts with c-Src in epithelial cells to control c-Src activity and morphological function [36]. The c-Src proto-oncogene has been strongly implicated in the development, growth, progression, and metastasis of a number of human cancers including those of the colon, breast, pancreas, and brain [37]. *PHLDA1* gene encodes Pleckstrin homology-like domain family A member 1 (PHLDA1 protein) that is involved in the regulation of apoptosis [38] and serves as a follicular stem cell marker [39]. PHLDA1 protein inhibits Akt and has tumor‐suppressive ability in breast and ovarian cancers [40]. *PRR21* (putative proline-rich protein 21, PRR21) is a single exon gene, previously annotated as “uncertain” by UniProtKB but since removed from the UniProtKB proteome [41]. GeneCards database summarizes diseases associated with *PRR21* including Bardet–Biedl Syndrome 4 and Peroxisome Biogenesis Disorder 2A. *GOLGA6L2* gene encodes golgin A6 family-like 2 [42]. Mutations in this gene have been reported in a breast cancer sample from The Cancer Genome Atlas project and in three patients with fibrolamellar hepatocellular carcinoma [43]. *KRTAP4-2* gene encodes keratin-associated protein 4–2. In the GeneCards database summary among its related pathways are keratinization and developmental biology. Keratin-associated proteins are the structural proteins of hair fibers and thought to play an important role in determining the physical properties of hair fibers [44]. These genetic alterations may play an important etiological role in teratoma formation.

The novel mutations in *DUSP5* and *PHLDA1* (bold in Fig. 4A) genes found on WES of mature cystic teratomas of the ovary may help to explain the presence of hair within these tumors. For example, the mutation p.Gln204del of the *PHLDA1* gene was observed in all teratomas in this study. The PHLDA1 protein is localized in the follicular bulge and acts as a stem cell marker of hair follicles [45]. Marchiori et al*.* also noted that estrogen can up-regulate *PHLDA1* transcription [46]. Further clarification of the mutant function on hair follicle may shed light on the treatment of diseases involving hair loss, e.g., alopecia. Another interesting finding in this study is the mutation p.Ala220Met of the *DUSP5* gene in all teratomas. DUSP5 protein plays an important role in the maintenance of stem cell pluripotency [32] and osteogenic differentiation [33]. Osteogenic differentiation is another characteristic of teratomas, i.e., bone or tooth formation. Further research on this mutant may point to a treatment method for diseases involving bone loss, e.g., osteoporosis.

## Conclusions

In summary, some important genes were identified in mature cystic teratomas of the ovary via WES in this study (Fig. 3). Mutations were validated in 15 genes with alterations in 9 samples (100%) and changes in protein coding (Fig. 3A, B). The top 10 mutated genes were *FLG*, *MUC17*, *MUC5B*, *RP1L1*, *NBPF1*, *GOLGA6L2*, *SLC29A3*, *SGK223*, *PTGFRN*, and *FAM186A*. Among the prevalent mutated genes, 7 variants in exons with changes in protein coding are important for the development of mature cystic teratomas of the ovary, including *PTGFRN*, *DUSP5*, *MPP2, PHLDA1*, *PRR21*, *GOLGA6L2,* and *KRTAP4-2*. These genetic alterations may play an important etiological role in teratoma formation. Moreover, novel mutations in *DUSP5* and *PHLDA1* genes found on WES may help to explain the characteristics of teratoma.

## Supplementary Information

**Additional file 1.** Genetic variants detected in exons with a change in protein coding in the top 10 mutated genes.

## Data Availability

The original data presented in the study are included in the article. Further inquiries can be directed to the corresponding author.

## Abbreviations

ACTG1: Actin Gamma 1
ADAM33: Disintegrin and metalloproteinase domain-containing protein 33
ATP5G1: ATP synthase lipid-binding protein
C2orf80: Chromosome 2 open reading frame 80
CACNA1A: Calcium voltage-gated channel subunit alpha1 A
CDKN2A: Cyclin-dependent kinase inhibitor 2A
CD9P-1: CD9 partner 1
DNA: Deoxyribonucleic acid
COSMIC: Catalogue of Somatic Mutations in Cancer
DUSP5: Dual specificity phosphatase 5
DBN1: Drebrin 1
DIP2B: Disco-interacting protein 2 homolog B
ERK: Extracellular signal-regulated kinase
EWI-F: EWI motif-containing protein F
FAM186A: Family with sequence similarity 186 member A
FLG: Filaggrin
FFPE: Formalin-fixed paraffin-embedded
GATK: Genome analysis toolkit
GATSL3: GATS-like protein 3
GOLGA6L2: Golgin A6 family-like 2
IFI27: Interferon alpha-inducible protein 27
ITGA6: Integrin subunit alpha 6
ITGB4: Integrin subunit beta 4
KRTAP4-2: Keratin-associated protein 4–2
MLH1: MutL homolog 1
LPAR1: Lysophosphatidic acid receptor 1
MMP17: Matrix metallopeptidase 17
MPP2: Membrane palmitoylated protein 2
MUC: Mucin
NBPF1: Neuroblastoma breakpoint family member 1
NEFH: Neurofilament heavy chain
NOP56: Nucleolar protein 56
OAS1: 2′-5′-Oligoadenylate synthetase 1
PHLDA1: Pleckstrin homology-like domain family A member 1
PIK3CA: Phosphatidylinositol-4,5-bisphosphate 3-kinase catalytic subunit alpha
POR: Cytochrome P450 oxidoreductase
PGF2-α: Prostaglandin F2-alpha
PRR21: Putative proline-rich protein 21
PTGFRN: Prostaglandin F2 receptor negative regulator
RP1L1: Retinitis pigmentosa-1-like-1
SCC: Squamous cell carcinoma
SCP1/2: Small c‐terminal phosphatase 1/2
SGK223: Sugen kinase 223
SLC29A3: Solute carrier family 29 member 3
SNVs: Single nucleotide variants
TP53: Tumor protein p53
XXYLT1: Xyloside xylosyltransferase 1
UV-VIS: Ultraviolet–visible
ZNF806: Zinc finger protein 806

## References

[1] Pantoja E, Noy MA, Axtmayer RW, Colon FE, Pelegrina I (1975). Ovarian dermoids and their complications. Comprehensive historical review. Obstet Gynecol Surv.

[2] Ingale Y, Shankar AA, Routray S, Agrawal M, Kadam A, Patil T (2013). Ectopic teeth in ovarian teratoma: a rare appearance. Case Rep Dent.

[3] Peterson CM, Buckley C, Holley S, Menias CO (2012). Teratomas: a multimodality review. Curr Probl Diagn Radiol.

[4] McKenney JK, Heerema-McKenney A, Rouse RV (2007). Extragonadal germ cell tumors: a review with emphasis on pathologic features, clinical prognostic variables, and differential diagnostic considerations. Adv Anat Pathol.

[5] Ellenson LH, Pirog EC. The female genital tract, 8th ed. Kumar V, Abbas AK, Fausto N, Aster JC, editors: Saunders; 2010.

[6] Wu RT, Torng PL, Chang DY, Chen CK, Chen RJ, Lin MC (1996). Mature cystic teratoma of the ovary: a clinicopathologic study of 283 cases. Zhonghua Yi Xue Za Zhi (Taipei).

[7] Lensch MW, Schlaeger TM, Zon LI, Daley GQ (2007). Teratoma formation assays with human embryonic stem cells: a rationale for one type of human-animal chimera. Cell Stem Cell.

[8] Blum B, Bar-Nur O, Golan-Lev T, Benvenisty N (2009). The anti-apoptotic gene survivin contributes to teratoma formation by human embryonic stem cells. Nat Biotechnol.

[9] Su W, Zhou M, Zheng Y, Fan Y, Wang L, Han Z (2011). Bioluminescence reporter gene imaging characterize human embryonic stem cell-derived teratoma formation. J Cell Biochem.

[10] Stachelscheid H, Wulf-Goldenberg A, Eckert K, Jensen J, Edsbagge J, Bjorquist P (2013). Teratoma formation of human embryonic stem cells in three-dimensional perfusion culture bioreactors. J Tissue Eng Regen Med.

[11] Przyborski SA (2005). Differentiation of human embryonic stem cells after transplantation in immune-deficient mice. Stem cells.

[12] Aleckovic M, Simon C (2008). Is teratoma formation in stem cell research a characterization tool or a window to developmental biology?. Reprod Biomed Online.

[13] Western PS, Ralli RA, Wakeling SI, Lo C, van den Bergen JA, Miles DC (2011). Mitotic arrest in teratoma susceptible fetal male germ cells. PLoS ONE.

[14] Tate G, Tajiri T, Suzuki T, Mitsuya T (2009). Mutations of the KIT gene and loss of heterozygosity of the PTEN region in a primary malignant melanoma arising from a mature cystic teratoma of the ovary. Cancer Genet Cytogenet.

[15] Li Y, Zhang R, Pan D, Huang B, Weng M, Nie X (2014). KRAS mutation in adenocarcinoma of the gastrointestinal type arising from a mature cystic teratoma of the ovary. J Ovarian Res.

[16] Cooke SL, Ennis D, Evers L, Dowson S, Chan MY, Paul J (2017). The driver mutational landscape of ovarian squamous cell carcinomas arising in mature cystic teratoma. Clin Cancer Res.

[17] Wang WC, Lai YC (2016). Genetic analysis results of mature cystic teratomas of the ovary in Taiwan disagree with the previous origin theory of this tumor. Hum Pathol.

[18] Wang WC, Lai YC (2017). Evidence of metachronous development of ovarian teratomas: a case report of bilateral mature cystic teratomas of the ovaries and systematic literature review. J Ovarian Res.

[19] Li H, Durbin R (2010). Fast and accurate long-read alignment with Burrows–Wheeler transform. Bioinformatics.

[20] McKenna A, Hanna M, Banks E, Sivachenko A, Cibulskis K, Kernytsky A (2010). The Genome Analysis Toolkit: a MapReduce framework for analyzing next-generation DNA sequencing data. Genome Res.

[21] McLaren W, Gil L, Hunt SE, Riat HS, Ritchie GR, Thormann A (2016). The ensembl variant effect predictor. Genome Biol.

[22] Alexandrov LB, Nik-Zainal S, Wedge DC, Aparicio SA, Behjati S, Biankin AV (2013). Signatures of mutational processes in human cancer. Nature.

[23] Davies H, Morganella S, Purdie CA, Jang SJ, Borgen E, Russnes H (2017). Whole-genome sequencing reveals breast cancers with mismatch repair deficiency. Can Res.

[24] Iwasa A, Oda Y, Kurihara S, Ohishi Y, Yasunaga M, Nishimura I (2008). Malignant transformation of mature cystic teratoma to squamous cell carcinoma involves altered expression of p53- and p16/Rb-dependent cell cycle regulator proteins. Pathol Int.

[25] Orlicky DJ, Nordeen SK (1996). Cloning, sequencing and proposed structure for a prostaglandin F2 alpha receptor regulatory protein. Prostaglandins Leukot Essent Fatty Acids.

[26] Orlicky DJ, Berry R, Sikela JM (1996). Human chromosome 1 localization of the gene for a prostaglandin F2alpha receptor negative regulatory protein. Hum Genet.

[27] Stipp CS, Orlicky D, Hemler ME (2001). FPRP, a major, highly stoichiometric, highly specific CD81- and CD9-associated protein. J Biol Chem.

[28] Charrin S, Le Naour F, Oualid M, Billard M, Faure G, Hanash SM (2001). The major CD9 and CD81 molecular partner. Identification and characterization of the complexes. J Biol Chem.

[29] Shan X, Yu T, Yan X, Wu J, Fan Y, Guan X (2021). Proteomic analysis of healthy and atretic porcine follicular granulosa cells. J Proteomics.

[30] Rong Wang CY, Fu L, Liu J, Li J, Yin L (2019). Expression profile analysis for epithelial-mesenchymal transition of breast cancer cell line DKTA based on microarray data. Eur J Gynaecol Oncol.

[31] Habibian JS, Jefic M, Bagchi RA, Lane RH, McKnight RA, McKinsey TA (2017). DUSP5 functions as a feedback regulator of TNFα-induced ERK1/2 dephosphorylation and inflammatory gene expression in adipocytes. Sci Rep.

[32] Chen Q, Zhou Y, Zhao X, Zhang M (2011). Effect of dual-specificity protein phosphatase 5 on pluripotency maintenance and differentiation of mouse embryonic stem cells. J Cell Biochem.

[33] Liu X, Liu X, Du Y, Hu M, Tian Y, Li Z (2021). DUSP5 promotes osteogenic differentiation through SCP1/2-dependent phosphorylation of SMAD1. Stem Cells.

[34] Mazoyer S, Gayther SA, Nagai MA, Smith SA, Dunning A, van Rensburg EJ (1995). A gene (DLG2) located at 17q12-q21 encodes a new homologue of the Drosophila tumor suppressor dIg-A. Genomics.

[35] Wang IC, Zhang Y, Snyder J, Sutherland MJ, Burhans MS, Shannon JM (2010). Increased expression of FoxM1 transcription factor in respiratory epithelium inhibits lung sacculation and causes Clara cell hyperplasia. Dev Biol.

[36] Baumgartner M, Weiss A, Fritzius T, Heinrich J, Moelling K (2009). The PDZ protein MPP2 interacts with c-Src in epithelial cells. Exp Cell Res.

[37] Irby RB, Yeatman TJ (2000). Role of Src expression and activation in human cancer. Oncogene.

[38] Park CG, Lee SY, Kandala G, Lee SY, Choi Y (1996). A novel gene product that couples TCR signaling to Fas(CD95) expression in activation-induced cell death. Immunity.

[39] Sellheyer K, Krahl D (2011). PHLDA1 (TDAG51) is a follicular stem cell marker and differentiates between morphoeic basal cell carcinoma and desmoplastic trichoepithelioma. Br J Dermatol.

[40] Chen Y, Takikawa M, Tsutsumi S, Yamaguchi Y, Okabe A, Shimada M (2018). PHLDA1, another PHLDA family protein that inhibits Akt. Cancer Sci.

[41] Abascal F, Juan D, Jungreis I, Kellis M, Martinez L, Rigau M (2018). Loose ends: almost one in five human genes still have unresolved coding status. Nucleic Acids Res.

[42] Jiang YH, Wauki K, Liu Q, Bressler J, Pan Y, Kashork CD (2008). Genomic analysis of the chromosome 15q11-q13 Prader–Willi syndrome region and characterization of transcripts for GOLGA8E and WHCD1L1 from the proximal breakpoint region. BMC Genomics.

[43] Darcy DG, Chiaroni-Clarke R, Murphy JM, Honeyman JN, Bhanot U, LaQuaglia MP (2015). The genomic landscape of fibrolamellar hepatocellular carcinoma: whole genome sequencing of ten patients. Oncotarget.

[44] Zhao Z, Liu G, Li X, Huang J, Xiao Y, Du X (2016). Characterization of the promoter regions of two sheep keratin-associated protein genes for hair cortex-specific expression. PLoS ONE.

[45] Ohyama M, Terunuma A, Tock CL, Radonovich MF, Pise-Masison CA, Hopping SB (2006). Characterization and isolation of stem cell-enriched human hair follicle bulge cells. J Clin Invest.

[46] Marchiori AC, Casolari DA, Nagai MA (2008). Transcriptional up-regulation of PHLDA1 by 17beta-estradiol in MCF-7 breast cancer cells. Braz J Med Biol Res.

